# Treatment for *Schistosoma japonicum*, Reduction of Intestinal Parasite Load, and Cognitive Test Score Improvements in School-Aged Children

**DOI:** 10.1371/journal.pntd.0001634

**Published:** 2012-05-01

**Authors:** Amara E. Ezeamama, Stephen T. McGarvey, Joseph Hogan, Kate L. Lapane, David C. Bellinger, Luz P. Acosta, Tjalling Leenstra, Remigio M. Olveda, Jonathan D. Kurtis, Jennifer F. Friedman

**Affiliations:** 1 Department of Nutrition, Harvard School of Public Health, Boston, Massachussetts, United States of America; 2 Department of Epidemiology, Brown University Medical School, Providence, Rhode Island, United States of America; 3 International Health Institute, Brown University, Providence, Rhode Island, United States of America; 4 Center for Statistical Sciences, Brown University, Providence, Rhode Island, United States of America; 5 Department of Epidemiology and Community Health, Virginia Commonwealth University, Richmond, Virginia, United States of America; 6 Department of Environmental Health, Children's Hospital, Boston, Massachussetts, United States of America; 7 Research Institute for Tropical Medicine, Manila, The Philippines; 8 Center for International Health Research (CIHR), Rhode Island Hospital (RIH), Providence, Rhode Island, United States of America; 9 Department of Pathology, Brown University Medical School, Providence, Rhode Island, United States of America; 10 Department of Pediatrics, Brown University Medical School, Providence, Rhode Island, United States of America; The George Washington University Medical Center, United States of America

## Abstract

**Background:**

To determine whether treatment of intestinal parasitic infections improves cognitive function in school-aged children, we examined changes in cognitive testscores over 18 months in relation to: (i) treatment-related *Schistosoma japonicum* intensity decline, (ii) spontaneous reduction of single soil-transmitted helminth (STH) species, and (iii) ≥2 STH infections among 253 *S. japonicum*-infected children.

**Methodology:**

Helminth infections were assessed at baseline and quarterly by the Kato-Katz method. *S. japonicum* infection was treated at baseline using praziquantel. An intensity-based indicator of lower *vs.* no change/higher infection was defined separately for each helminth species and joint intensity declines of ≥2 STH species. In addition, *S. japonicum* infection-free duration was defined in four categories based on time of schistosome re-infection: >18 (i.e. cured), >12 to ≤18, 6 to ≤12 and ≤6 (persistently infected) months. There was no baseline treatment for STHs but their intensity varied possibly due to spontaneous infection clearance/acquisition. Four cognitive tests were administered at baseline, 6, 12, and 18 months following *S. japonicum* treatment: learning and memory domains of Wide Range Assessment of Memory and Learning (WRAML), verbal fluency (VF), and Philippine nonverbal intelligence test (PNIT). Linear regression models were used to relate changes in respective infections to test performance with adjustment for sociodemographic confounders and coincident helminth infections.

**Principal Findings:**

Children cured (β = 5.8; P = 0.02) and those schistosome-free for >12 months (β = 1.5; P = 0.03) scored higher in WRAML memory and VF tests compared to persistently infected children independent of STH infections. A decline *vs.* no change/increase of any individual STH species (β:11.5–14.5; all P<0.01) and the joint decline of ≥2 STH (β = 13.1; P = 0.01) species were associated with higher scores in WRAML learning test independent of schistosome infection. Hookworm and *Trichuris trichiura* declines were independently associated with improvements in WRAML memory scores as was the joint decline in ≥2 STH species. Baseline coinfection by ≥2 STH species was associated with low PNIT scores (β = −1.9; P = 0.04).

**Conclusion/Significance:**

Children cured/*S. japonicum*-free for >12 months post-treatment and those who experienced declines of ≥2 STH species scored higher in three of four cognitive tests. Our result suggests that sustained deworming and simultaneous control for schistosome and STH infections could improve children's ability to take advantage of educational opportunities in helminth-endemic regions.

## Author Summary

Parasitic worm infections are associated with cognitive impairment and lower academic achievement for infected relative to uninfected children. However, it is unclear whether curing or reducing worm infection intensity improves child cognitive function. We examined the independent associations between: (i) *Schistosoma japonicum* infection-free duration, (ii) declines in single helminth species, and (iii) joint declines of ≥2 soil-transmitted helminth (STH) infections and improvements in four cognitive tests during18 months of follow-up. Enrolled were schistosome-infected school-aged children among whom coinfection with STH was common. All children were treated for schistosome infection only at enrolment with praziquantel. Children cured or schistosome-free for >12 months scored higher in memory and verbal fluency tests compared to persistently infected children. Likewise, declines of single and polyparasitic STH infections predicted higher scores in three of four tests. We conclude that reducing the intensity of certain helminth species and the frequency of multi-species STH infections may have long-term benefits for affected children's cognitive performance. The rapidity of schistosome re-infection and the ubiquity of concurrent multi-species infection highlight the importance of sustained deworming for both schistosome and STH infections to enhance the learning and educational attainment of children in helminth-endemic settings.

## Introduction

Many children in developing countries perform below academically desired levels [1]. Helminth infections are a pervasive part of children's environments in these settings that may contribute to poor educational outcomes through reduced iron status, inflammation, decreased macro-nutrient nutritional status, and distracting symptoms such as abdominal pain [2], [3].

Some epidemiologic studies have linked these infections to low academic achievement in resource-limited settings [4]–[7]. However, many of the studies did not control for important confounders or had methodological differences that made comparability of findings across studies difficult [8]. All but two prior studies [9], [10] examined associations between cognitive performance and single helminth species. Recently, polyparasitism, that is, the concurrent multi-species helminth infection, has been associated with childhood anemia and self-reported morbidity [11]–[13]. Its relationship to performance in cognitive tests deserves specific investigation [8].

An earlier cross-sectional study by our group found that moderate or higher intensity infection with *Trichuris trichiura, Ascaris lumbricoides*, and *Schistosoma japonicum* were, respectively, associated with low scores on tests of verbal fluency, and the memory and learning subscales of the Wide Range Assessment of Memory and Learning (WRAML) tests in school-aged children [14]. It is expected that treatment for parasitic helminth infections will confer a range of benefits to child health, including improvements in academic performance among heavily infected children [15]. However, empirical support for this claim is lacking [8]. Short follow-up periods for most randomized controlled trials, variability in prevalence and baseline intensities of helminth infections, and a background of high re-infection pressure could explain failure to consistently find treatment-associated score improvements.

The ambiguity in the literature justifies further exploration of this subject and motivates this longitudinal study to determine the relationship between cognitive testscore improvement and independent declines of schistosome and single soil-transmitted helminth (STH) infections, as well as the impact of concurrent declines of two or more STHs on changes in cognitive testscores. Specifically, we provide associations between cognitive testscore improvement and: (i) treatment-induced changes in *S. japonicum* intensity, (ii) non-treatment-related or natural declines in single STH infections, and (iii) joint infection decline for ≥2 STH species. We hypothesize that no or low level *S. japonicum* re-infection after praziquantel treatment, and clearance or intensity reductions for single and polyparasitic STH infections will predict improvements in cognitive testscores during follow-up among school-aged children living in a schistosome and STH co-endemic area of Leyte, The Philippines.

## Materials and Methods

### Ethics Statement

The parent study and the nested study reported here were approved by the Brown University, Lifespan, and Philippines Research Institute of Tropical Medicine Institutional Review Boards. Participants' aged ≥18 years provided written informed consent. In addition, all parents/guardians provided written informed consent on behalf of child participants, whereas children aged ≥8 years provided assent. All participants were *S. japonicum* infected and were treated with the anti-schistosomal drug praziquantel (60 mg/kg over 4 hours) at enrolment as part of the parent study. Only cognitive testing was conducted in a subset of 253 children, aged 7–19 years, as part of this nested observational study.

There was no baseline treatment for STH infections as large-scale helminth treatment campaigns were not available in The Philippines at the time this study was conducted. However, at the end of the study, children with STH infection were treated with albendazole and those that became re-infected with *S. japonicum* were treated with praziquantel. An approach that includes waiting to treat children infected with STH would not be taken today given more recent published findings regarding subtle morbidities related to STH infections.

### Study Design and Population

This study was conducted in Macanip, a malaria-free rural rice farming village in Leyte, The Philippines, where *S. japonicum* and STH infections coexist with high prevalence. This is a nested prospective cohort study conducted in a subset of *S. japonicu*- infected Filipinos aged 7–30 years who were enrolled in a study of immune correlates of resistance to *S. japonicum* reinfection [16].

Eligibility criteria included: baseline *S. japonicum* infection, age 7–19 years at enrolment, provision of parental consent, and child assent for participation in this study. Exclusion criteria included pregnancy or lactation, severe malnutrition (weight-for-height z-score<−3), severe anemia (hemoglobin<7 g/dl), or the presence of a serious chronic disease determined by history, physical examination, or laboratory findings.

### Outcome Assessment: Cognitive Tests

Four cognitive tests were administered, including the Philippine nonverbal intelligence test (PNIT), verbal fluency (VF), and two domains of the Wide Range Assessment of Learning and Memory (WRAML), namely verbal memory and learning. Tests were chosen based on their ability to capture a range of cognitive processes including fluid intelligence (PNIT), learning (WRAML), and memory (VF and WRAML) while being adaptable across different cultures. The PNIT is an intelligence test that measures concept recognition and abstract thinking [17]. VF test is thought to be a good measure of the central executive component of working memory. The WRAML assesses a child's ability to learn and recall new information. Specifically, the WRAML learning subtests evaluate a child's performance over trials on tasks using the free-recall paradigm, while the WRAML verbal memory subtests assess a child's memory capabilities on meaningful (i.e., stories) and meaningless material (i.e., strings of random digits and letters) [18]. Each of the domains assessed by the WRAML consists of three age-standardized subtests that are added together to derive a total age- and gender-scaled score per domain. Unlike the WRAML, neither the PNIT nor the VF are age standardized; therefore, these tests were adjusted for age variation using linear regression from which we calculated the error terms associated with each child's testscore. We then modeled as the dependent variable the error terms associated with performance in PNIT and VF tests.

All tests were translated, adapted for cultural appropriateness, and pilot tested among Filipino children from other *S. japonicum*-endemic villages near the study area. Testing was conducted in a designated room adjacent to the field laboratory with sufficient lighting and minimal external noises. Ambient temperature within the classroom was approximately 27°C. All children were provided a snack about 30 minutes prior to testing.

Joint inter-rater and test-retest reliability with a 6-week interval between tests were evaluated. Cronbach's alpha coefficient was used to assess the degree of internal consistency between tests in the WRAML learning (α = 0.54) and WRAML verbal memory (α = 0.81) domains. For all tests, higher scores correspond to better performance. Details of each test and its psychometric properties have been previously reported [14]. More details about the rationale for choosing specific tests and their respective properties are presented in Appendices S1 and S2.

Cognitive assessments were made at months 0, 6, 12, and 18. All infections were assessed at baseline and quarterly thereafter. We have previously reported on cross-sectional associations between helminth infections and performance in the aforementioned tests [14]. Here we determine associations between post-treatment testscores and: (i) post-treatment re-infection with *S. japonicum* and (ii) natural infection clearance/decline for STH infections. Only cognitive assessments at 6, 12, and 18 months are included in the outcome matrix to preserve temporal sequence between infections and testscore changes.

### Helminth Infections

The origin of this prospective analysis is the cohort-wide interval of least infection intensity for all species (i.e., months 1–3). STH and schistosome infections were assessed at months 0, 3, 6, 9, 12, 15, and 18. For *S. japonicum* only, an additional assessment (one month post-treatment) was done to evaluate treatment efficacy.

The number of eggs per gram (EPG) of stool was determined via duplicate examination of three stool samples by the Kato-Katz method for all species [19]. EPGs were used to define none, low, moderate, or high intensity categories for each species using World Health Organization EPG thresholds [20]. For each individual helminth species, except hookworm, a separate dichotomous baseline intensity indicator was defined as: uninfected/low *vs.* moderate/high infection to accommodate the intensity distribution in this cohort. For hookworm infection only, baseline infection intensity was defined as none *vs.* any infection, since >40% of participants were hookworm-free at enrollment and those infected had predominantly low infections.

### Baseline Polyparasitic STH Infections

Children were initially grouped by the intensity of concurrent infection with hookworm, *A. lumbricoides* and *T. trichiura* as having: (i) one or zero low; (ii) two or three low; (iii) one moderate/high STH; (iv) two moderate/high; and (v) three moderate/high intensity coinfections [11]. These categories were further combined into one baseline polyparasitic STH indicator to distinguish children with ≥2 STH species at moderate/high intensity (which may include zero or one low infection of the third STH species) from those with at most one STH infection at moderate or higher intensity STH coinfection (other STHs are either absent or present at low intensity only).

### Infection Change for Single Helminth Species

Given our treatment-reinfection design and study inclusion predicated on *S. japonicum* infection, the most dynamic infection changes occurred with respect to *S. japonicum* during follow-up; however, STH infection intensity also varied over time. These non-treatment related changes in STH intensity may be due to one or more of the following factors: (i) natural changes in STH infections within individuals over time, (ii) the limited sensitivity of some STH species to praziquantel [21], [22], and (iii) lower diagnostic sensitivity for the Kato-Katz method especially when used for the simultaneous assessment of multiple STH species at low intensity in the same host [23]. We defined three post-treatment infection intervals: 1≤t_1_<6, 6≤t_2_≤12 and 12<t_3_≤18 months; to correspond with the three repeated cognitive assessments. For each STH, t_1_ infection value (I_1_) was the mean EPG at month three, whereas for *S. japonicum* I_1_ was the mean of EPGs at months one and three. T_2_ infection (I_2_) was the mean of EPGs at months six and nine, and t_3_ infection (I_3_) was the mean of EPGs at months 12, 15, and 18. Within respective intervals, intra-individual infection change scores (δ_it_) were defined by species as follows: t_2_: δ_i2_ = I_i2_ - I_i1_; and t_3_: δ_i3_ = I_i3_ - I_i1_.

Hence, δ_it_ ranged from −∞ to +∞ and will be negative, zero, or positive for a given STH species if the child's infection was lower, equivalent to, or greater than their infection intensity at t_1_. For each species, separate δ_it_ values were defined and ultimately dichotomized into high *vs.* low categories as δ_it_≥0 *vs* δ_it_<0.

For *S. japonicum* only, infection-free duration was defined as a four level categorical variable that is: (i) 0 if not reinfected by month 18; (ii) 1 if reinfected between months 12 and 18; (iii) 2 if reinfected between months 6 and 12; and (iv) 3 if never cured or *S. japonicum* positive in t_1_, t_2_, and t_3_ (reference group). Children reinfected by 6, 12, or 18 months were compared to those not reinfected by study end.

### Infection Change for Polyparasitic STH Infections

We determined the number of concurrent STH declines as the sum of individual STH intensity declines using the previously described dichotomous infection decline variable based on δ_it_. Possible values for polyparasitic STH declines were: 0 = no decline/increase STH species, 1 = any one STH, 2 = any two STH to 3 = all STH species intensity decline in a given interval. Using these values, polyparasitic STH decline within intervals was defined as: concurrent intensity decline of ≥2 *vs.* ≤1 of 3 STH species.

### Confounders

We considered an extensive array of potential confounding factors. Because exposure to helminth infection and cognitive testscores vary by age, sex, and socioeconomic status (SES), these factors were considered non-time varying potential confounders. SES measurements were based on baseline questionnaire data addressing four domains of social position; parental and child education, occupation, home/land ownership, and assets. The method used to derive and validate this measure of SES has been described elsewhere [14], [24]. The derived summary SES variable is divided into four ordinal categories by the quartiles of its distribution.

Anemia and nutritional status at baseline were considered potential confounders and/or mediators of low testscores. Anemia was defined on the basis of age- and sex-specific hemoglobin cutoffs recommended by the WHO [25]. Hemoglobin measurement was based on complete hemograms determined on a Serono Baker 9000 hematology analyzer (Serono Baker Diagnostics, Allentown, PA). Nutritional status was assessed using weight-for-age z-scores (WAZ) calculated using the National Center for Health Statistics year 2000 reference values in EpiInfo software (version 2000, Atlanta, GA). Normal and malnutrition status were defined by WAZ≥−2 and WAZ<−2, respectively.

### Statistical Analysis

Multivariable random effects regression models were fitted separately to each cognitive test without adjusting for testscore at study enrollment (month 0) given our observational study design [26]. We assumed an unstructured covariance matrix to account for non-independence of repeated cognitive tests within individuals and accounted for clustering of observations within households by including a random intercept for household. Empirical standard errors were used for all estimations to ensure that significance tests were robust against mis-specification of the covariance matrix.

In addition, we examined the relationship between test performance and *S. japonicum*-free duration in separate regression models. Sample regression models for estimation of associations between testscores and *S. japonicum* infection decline and *S. japonicum* infection free duration are provided in Appendix S3.

Finally, we examined the potential for modification in the association between infection change and testscore improvement by the following baseline factors: helminth infection intensity, underweight, and anemia. For example, to examine whether the relationship between hookworm infection decline and testscore improvement was heterogenious by hookworm baseline infection intensity, we introduced a three-way multiplicative interaction consisting of the dichotmous indicator of hookworm infection decline, time, and baseline hookworm intensity in a multivariate models that in addition to other confounders also adjust for the baseline intensity of *A. lumbricoides, T. trichiura* and *S. japonicum* as well as each of the three dichotmous indicators of change in these infections from the interval of lowest infection. We then examined the p-values associated with interaction terms and where P≤0.05, results are presented by strata of baseline hookworm intensity. The same approach was used to examine baseline underweight and baseline anemia as potential effect modifiers in separate multivariate regression models.

## Results

The prevalence of *A. lumbricoides, T. trichiura* and hookworm infections in this *S. japonicum*-infectected cohort at baseline were 79.9%, 95.6%, and 50.6%, respectively. Of the 253 children, 97% were concurrently infected by *S. japonicum* and at least one STH species, approximately 36% were anemic and 60% were underweight relative to U.S. children of the same age and sex (Table 1).

**Table 1:** Baseline sociodemographic characteristics of *S. japonicum*-infected school-aged children enrolled between 2002 and 2003 in Leyte, The Philippines.

Characteristic	N (%)
Sex	
Male	147 (58.1)
Female	106 (41.9)
Age (years)	
7–9	63 (24.9)
10–12	47 (18.6)
13–16	99 (39.1)
16–19	44 (17.4)
High socioeconomic status	126 (49.8)
Nutritional status	
Underweight (WAZ<−2)	147 (59.8%); missing = 7
Stunting (HAZ<−2)	165 (66.8); missing = 6
*Schistosoma japonicum* intensity	N = 253
Uninfected	0 (0%)
Low (1–99 EPG)*	177 (70.0%)
Moderate (>99–399 EPG)	57 (22.5%)
High (>399 EPG)	19 (7.5%)
Mean EPG (SD)	120 (198)
Median EPG (IQR)*	40 (110)
*Ascaris lumbricoides* intensity**	N = 249
Uninfected	50 (20.08%)
Low (1–4999 EPG)	62 (25.0%)
Moderate (>4999–49,999 EPG)	102 (41.0%)
High (>49,999 EPG)	35 (14.1%)
Mean EPG (SD)	21,344 (33,801)
Median EPG (IQR)	9067 (24,727)
*Trichuris trichiura* intensity**	N = 249
Uninfected	11 (4.4%)
Low (1–999 EPG)	95 (38.2%)
Moderate (>999–9,999 EPG)	125 (50.2%)
High (>9,999 EPG)	18 (7.2%)
Mean EPG (SD)	3267 (8282)
Median EPG (IQR)	1320 (2312)
Hookworm intensity**	N = 249
Uninfected	123 (49.4%)
Low (1–1999 EPG)	118 (47.4%)
Moderate/High (>1999 EPG)	8 (3.20%)
Mean EPG (SD)	293 (680)
Median EPG (IQR)	6.7 (280)
Helminth co-infection frequency***	N = 249
None/1 low intensity	19 (7.6%)
2/3 low intensity only	42 (16.9%)
1 Moderate/high intensity	33 (13.3%)
2 Moderate/high intensity	36 (14.5%)
3 Moderate/high intensity	119 (47.8%)
Cognitive tests scores	N
	Mean (SD)
WRAML learning index score	N = 241
	80.9 (14.6)
WRAML verbal index score	N = 231
	58.6 (11.8)
Fluency score	N = 241
	18.1 (4.9)
Philippine nonverbal intelligence test	N = 241
	28.3 (7.4)
Hemoglobin (g/dl)	N = 241
	12.0 (1.6)

*: WRAML = Wide Range Assessment of Memory and Learning; EPG = eggs per gram of stool; IQR = inter-quartile range, SD = standard deviation.

**: assessment of STH infections were missing for four children throughout the study period.

***: Co-infection frequency includes both soil-transmitted helminths and *Schistosoma japonicum*.

**Figure 1:**
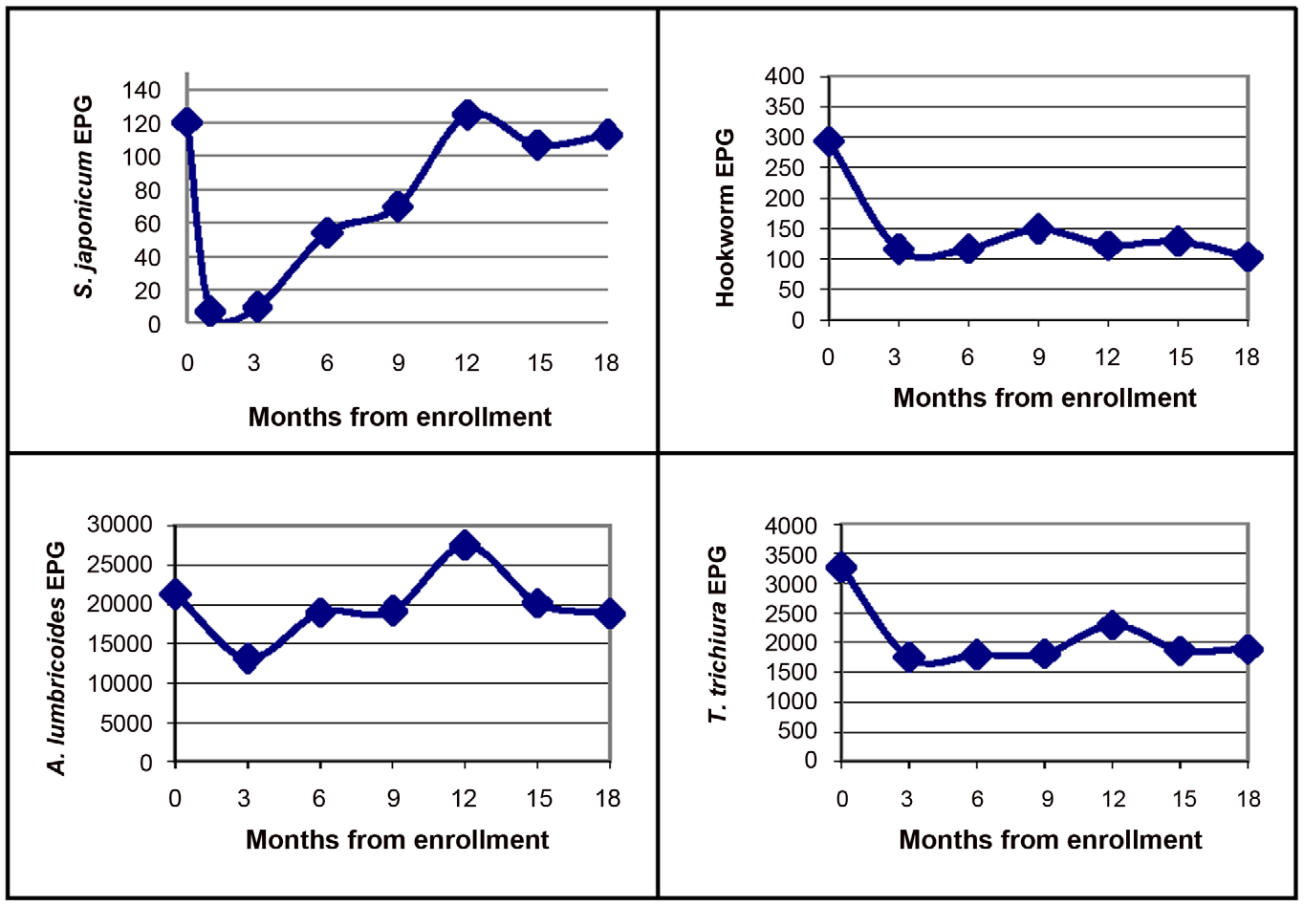
Cohort-wide variation in average infection intensity from enrolment through 18 months of follow-up in children aged 7–19 years from in Leyte, The Philippines. On the y-axis are plotted number of parasite eggs per gram (EPG) of stool and the x-axis shows the number of months from enrollment (time point zero) through 18 months. Study was implemented between 2002 and 2004 and included children aged 7–19 years at enrolment, residing in Leyte, The Philippines.

The lowest intensity of *S. japonicum* infection (mean = 6.8 EPG) occurred one month post-treatment at which 92% (n = 217) of the sample was infection-free. However, re-infection was rapid and increased steadily until the 12^th^ month of follow-up, at which point 70.8% of participants were infected with *S. japonicum*. Only 25 (10.6%) of the re-examined children were free of *S. japonicum* infection at 18 months. Individual STH intensities also declined from enrollment with the lowest average infection for all STH species occurring at three months. Infection intensity stabilized near this level throughout follow-up for hookworm and *T. trichiura* infections. The cohort-wide, *A. lumbricoides* infection intensity by the 18^th^ month was comparable to month zero despite the initial decline post-*S. japonicum* treatment (Figure 1).

From multivariable models adjusted for sociodemographic characteristics and the intensity of coincident *S. japonicum* and STH species, declines in the intensity of *T. trichiura*, hookworm, and polyparasitic STH infections were independently associated with higher average scores on the learning and verbal memory domains of WRAML tests during follow-up. Similarly, *A. lumbricoides* intensity decline was independently associated with higher scores in the learning sub-scale of WRAML. The intensity of individual infections at enrollment were generally not associated with performance on any of the tests employed, except for moderate/high intensity polyparasitic STH infection, which was associated with lower scores on the PNIT (Table 2).

**Table 2:** Test score change over 18 months follow-up in relation to single and polyparasitic helminth infection decline among school-aged children from Leyte, The Philippines.*

	WRAML test -learning subscale	WRAML test - memory subscale	Verbal fluency test	Philippine von-verbal intelligence test
Baseline infection intensity	Testscore difference (95% CI)	Testscore difference (95% CI)	Testscore difference (95% CI)	Testscore difference (95% CI)
Moderate/high (>99 EPG) *S. japonicum* **	−1.17 (−4.83, 2.48)	1.65 (−1.33, 4.63)	−0.89 (−2.10, 0.31)	−0.34 (−1.95, 1.27)
Moderate/high (>4999 EPG) *A. lumbricoides*	−1.34 (−4.78, 2.10)	1.04 (−1.72, 3.81)	0.14 (−1.00, 1.28)	−0.78 (−2.20, 0.65)
Moderate/high (>999 EPG) *T. trichiura*	1.28 (−2.06, 4.62)	−1.24 (−4.20, 1.20)	0.14 (−0.97, 1.24)	−0.77 (−2.33, 0.78)
Any (≥1 EPG) hookworm	1.28 (−2.27, 4.83)	1.09 (−2.00, 4.18)	0.24 (−0.81, 1.28)	−0.25 (−1.96, 1.46)
≥2 Moderate/high intensity STH infections	−1.20 (−5.72, 3.32)	−1.46 (−4.00, 1.97)	−0.14 (−1.80, 1.52)	**−1.92 (−3.70, −0.13)**
**Decline in infection intensity**				
*S. japonicum*	2.80 (−4.22, 10.89)	4.92 (−9.76, 19.60)	1.86 (−4.13, 7.85)	2.06 (−3.17, 7.29)
*A. lumbricoides*	**13.53 (5.80, 21.25)**	4.35 (−2.50, 11.20)	2.99 (−0.47, 6.46)	−1.65 (−5.48, 2.49)
*T. trichiura*	**11.51 (3.39, 19.63)**	**5.13 (0.32, 9.94)**	0.68 (−2.21, 3.58)	−1.24 (−3.81, 1.33)
Hookworm	**14.52 (4.35, 24.68)**	**9.77 (3.14, 16.40)**	3.09 (−1.89, 8.08)	2.35 (−1.96, 6.66)
Polyparasitic (≥2 species) STH intensity decline	**13.12 (4.37, 21.87)**	**10.27 (5.05, 15.48)**	2.03 (−1.98, 6.04)	0.15 (−3.30, 3.61)
**Other covariates**				
Time from enrollment	3.55 (2.22, 4.88)	2.83 (1.79, 3.88)	−1.81 (−2.38, −1.23)	−0.18 (−1.07, 0.71)
Age at enrollment	−3.34 (−4.75, −1.94)	−2.22 (−3.35, −1.09)	1.56 (0.95, 2.18)	−0.01 (−0.86, 0.77)
Anemia at enrollment	−2.27 (−6.18, 1.63)	−2.86 (−5.93, 0.20)	−1.08 (−2.32, 0.15)	−1.39 (−3.13, 0.34)
Underweight at enrollment (WAZ≤−2 *vs.*WAZ>−2)	−0.90 (−4.67, 2.87)	−0.56 (−3.43, 2.31)	−0.33 (−1.43, 0.77)	−0.33 (−1.67, 1.02)

Children were recruited between 2002 and 2004. WRAML = Wide Range Assessment of Memory and Learning; CI = confidence interval; EPG = eggs per gram of stool; STH = soil-transmitted helminth infections; WAZ = weight-for-age z-scores.

*: Estimates are differences in testscores for declines *vs.* no change or increase in helminth infections and other covariates shown. Estimated are derived a repeated measures linear mixed model adjusted for: time, child age at enrollment, sex, nutritional status, baseline *S. japonicum* infection, baseline STH intensity, socioeconomic status, and baseline anemia.

**: The reference group for baseline *S. japonicum* intensity consists of low intensity infections. For *A. lumbricoides* and *T. trichiura* baseline infections, the reference group consists of children with none/low infections. For hookworm infection only, the reference group consists of uninfected children. For baseline polyparasitic STH infections, the reference group includes children with none/all low infections.

A decline *vs.* no change or an increase in *S. japonicum* intensity from the interval of least infection was not independently associated with improvements in any tests over the study period (Table 2). We found no evidence that the relationship between *S. japonicum* infection decline and performance in respective tests differed within strata of *S. japonicum* intensity at enrollment (data not shown). However children who were *S. japonicum* free for ≥18 months or those who were *S. japonicum* infection free until 12 months post-treatment scored higher in all tests relative to rapidly re-infected or persistently infected children. The strength of association was generally attenuated in multi-variable models that controlled for several sociodemographic characteristics and coincident STH and the baseline intensity of *S. japonicum* infection. Nevertheless, never *S. japonicum* re-infected children and those *S. japonicum* infection-free for up to 12 months scored higher in the verbal memory sub-scale of WRAML and VF test, respectively (Table 3).

**Table 3:** Change in cognitive testscores over 18 months follow-up in relation to *S. japonicum* infection-free duration in Filipino school-aged children treated for *S. japonicum* at enrolment.

	WRAML test - learning subscale		WRAML test - memory subscale		Verbal fluency test		Philippine non-verbal intelligence test	
	Univariate association Score difference (95% CI)	Adjusted* association Score difference (95% CI)	Univariate association Score difference (95% CI)	Adjusted association Score difference (95% CI)	Univariate association Score difference (95% CI)	Adjusted association Score difference (95% CI)	Univariate association Score difference (95% CI)	Adjusted association Score difference (95% CI)
Not reinfected by 18 months	7.74	4.87	8.93	5.78	4.26	1.77	1.10	−0.76
(n = 25)	(−0.28, 15.8)	(−4.03, 13.8)	(3.73, 14.13)	(0.88, 10.68)	(1.01, 7.50)	(−0.56, 5.11)	(−1.51,3.71)	(−3.52, 2.02)
Reinfected between months 12 and 18	6.44	2.31	5.39	3.44	2.89	1.46	2.68	1.57
(n = 49)	(0.73, 12.09)	(−3.77, 8.39)	(0.87, 9.90)	(−0.86, 7.75)	(1.56, 4.21)	(0.07, 2.84)	(−0.63, 4.73)	(−0.63, 3.75)
Reinfected between months 6 and 12	1.01	0.58	−0.59	−0.67	0.91	0.54	0.72	0.56
(n = 104)	(−3.38, 5.39)	(−3.83, 4.99)	(−3.94, 2.76)	(−4.15, 2.80)	(−0.39,2.21)	(−0.66, 1.74)	(−1.19,2.63)	(−1.35, 2.47)
Rapidly reinfected (n = 75)**	Reference	Reference	Reference	Reference	Reference	Reference	Reference	Reference

WRAML = Wide Range Assessment of Memory and Learning; CI = confidence interval.

*: Estimates are differences in testscores for varying durations of *S. japonicum* cure relative to never cured children. Estimated are derived from a repeated measures model adjusted for: time, child age, sex, nutritional status, baseline *S. japonicum* infection, baseline STH intensity, socioeconomic status, and baseline anemia.

**: This category includes children that were *S. japonicum* infected during all periods for which infection was assessed. Some children may not have assessments in all intervals but if they were always *S. japonicum* positive when infection data is available they are considered never cured.

Anemia and underweight status at enrollment were not independently associated with performance in any tests. However, among children with anemia at enrollment, *S. japonicum* decline was associated with higher scores on WRAML learning subscale (mean = 10.5, 95% confidence interval (CI): 4.8–16.3). There was no association between *S. japonicum* infection decline and performance in WRAML learning subscale among children without anemia at enrollment (mean = −3.0, 95% CI: −6.4–0.4).

## Discussion

In this cohort of *S. japonicum*-infected children whose infections were treated at enrollment, we found positive associations between performance in the verbal memory subscale of WRAML and the test of verbal fluency and longer *S. japonicum* infection-free duration independent of concurrent STH infections. We also found parasite decline-associated improvements for scores in the learning and memory subscales of WRAML; specifically scores for these tests improved for children whose hookworm, *T. trichiura*, and polyparasitic STH infections declined relative to those who experienced no change or an increase in these infections from the interval of lowest infection. Further, declining *A. lumbricoides* was independently associated with superior testscores in the learning subscale of the WRAML. With the exception of baseline moderate or high intensity polyparasitic STH infection, which was associated with low PNIT scores, baseline helminth infection intensities were not generally associated with testscores over the study period.

Our finding that *S. japonicum* infection-free duration was associated with higher testscores in WRAML verbal memory subscale and verbal fluency test corroborates similar findings from a randomized controlled trial of *S. japonicum* using a different battery of tests in a subset of young children from the People's Republic of China [27]. Similarly, the positive associations between declines in polyparasitic STH and performance in the WRAML tests corroborates finding from two cross-sectional studies conducted among children from South Africa and Brazil [9], [10]. In the South African study, children with intestinal parasites and *S. mansoni* scored significantly lower on tests of sustained attention compared to uninfected children or children with single species infections [10]. More recently, Brazilian children concurrently infected with hookworm and *A. lumbricoides* scored lower on a different battery of cognitive tests relative to children with only single infections [9].

With respect to individual infections, we show that longer *S. japonicum* infection-free duration predicted significantly higher testscores in WRAML verbal memory independent of the intensity of coincident STH infections. Likewise declines in *A. lumbricoides* and *T. trichiura* intensities were independently associated with improvements WRAML learning score. These findings are congruent with our previously published cross-sectional findings in this cohort that these infections were associated with lower cross-sectional scores in both subscales of WRAML and the verbal fluency test [14]. In addition, our finding that decline in *T. trichiura* intensity over time was associated with significant elevation in both subscales of WRAML scores is in agreement with prior observations among helminth infected Ecuadorian [28] and East African [4] children. Our finding of positive associations between hookworm infection declines and improvements in both WRAML tests is supported by recent hookworm-associated cross-sectional report of lower concentration and information processing in Brazilian children [9].

In line with our hypothesis, baseline moderate/high intensity polyparasitic STH infections predicted lower average score on the PNIT. Further, a significant improvement in WRAML verbal memory testscores was evident for children with anemia but not for children who were not anemic at enrolment. These observations suggest that: (i) helminth infections combine with other infections, hematologic and nutritional risk factors to impair cognitive performance, and (ii) the cognitive benefit of declines in helminth infection intensity may be blunted in some subgroups depending on the extent of anemia, malnutrition and other infection they start out with. Nevertheless, we believe that all children in helminth endemic areas will likely benefit from a multi-pronged control strategy, including sustained deworming and improvement of nutritional status in the effort to counteract the effects of helminth infections on academic performance [29], [30]. These interactions may also explain some of the controversial findings in the literature as treatment benefits may be more profound in certain sub-groups, which, if not explored, may lead to different interpretations.

To put into context the improvements observed in this study with declines in various individual species or polyparasitic STH infections, and to evaluate their public health relevance, we compared our estimates to differences in cognitive test performance for children exposed to well known risk factors of cognitive impairment in children – including malaria [31] and fetal alcohol exposure [32]. Our estimated improvements in WRAML learning tests scores associated with single and polyparasitic STH intensity declines over the 18 months of this study is 6.3–7.8 and 7.1–8.9 times the improvement observed when African children without a history of hospitalization for severe or cerebral malaria were compared to children with severe or cerebral malaria infection using a different battery of tests [31]. Likewise, the WRAML verbal memory score differential for *S. japonicum* cured *vs.* never cured children is approximately 3.7 times the difference in performance reported 6–10 years later for a cohort of American children free of prenatal alcohol exposure relative to children exposed to these substances *in-utero* using the same tests [32]. Hence we conclude that the cognitive improvements noted with infection decline here are at least comparable to those associated with other well known important determinants of pediatric cognitive impairment and are therefore likely to be of clinical and public health relevance.

*S. japonicum*, *A. lumbricoides*, *T. trichiura*, and polyparasitic STH infections may impair children's performance in cognitive tests through adverse effects on iron and nutritional deficiencies associated with the presence of these parasites [3]. In addition, cytokines made in response to infection, particularly *S. japonicum* [33]–[36], which lives in the bloodstream, may have direct adverse effects on cognitive processing. Interferon gamma (IFN-gamma) and TNF-alpha, are thought to mediate “sickness behavior” [37], which refers to the behavioral, neurological, and cognitive alterations described in various infectious and inflammatory disease states [38]. Human studies have specifically related elevated levels of TNF-alpha and IFN-gamma to dysfunction in the memory domain [39], [40] and other work in this cohort suggests that anemia of inflammation may be an important contributor to congnitive impairment [41].

### Limitations and Strengths

Given our observational study design, we cannot exclude residual confounding by unmeasured covariates as an alternative explanation for our findings. By comparing children present at 18-months with those present at baseline on key factors, children scoring in the highest tertile of WRAML verbal memory at baseline and girls were over-represented among those lost to follow-up; however, there was no difference in average hemoglobin, SES, baseline STH intensity and average scores in WRAML learning, PNIT, and VF. In addition, the Kato-Katz relative to other helminth diagnostic methods has been reported to be of lower sensitivity for detecting helminth eggs particularly for individuals with light infections [42] and those with concurrent multi-species infections [23]. We expect that our duplicate assessment of three separate stool samples for each child would have improved the accuracy of helminth diagnosis in this study; however, we are unable to rule out the possible impact of limited sensitivity for lightly infected children.

To our knowledge, this is the first longitudinal study to investigate the independent effect of schistosome and individual STH infections as well as that of polyparasitic STH infection decline on learning domains of cognitive function, which may better reflect children's ability to take advantage of limited educational opportunities. The prospective study design, control for coincident helminth infections and numerous other confounders, and the explicit exploration of baseline infection, anemia and nutritional statuses as potential mediators of observed associations are additional strengths of this study.

We observed notable fluctuations in *T. trichiura* and *A. lumbricoides* intensity in this study even though only *S. japonicum* infection was treated at enrolment. Praziquantel, however, has been shown to have some anti-hookworm activity [22]. Unlike prior investigations of this question, our analytic strategy highlights the cognitive performance deficits associated with *S. japonicum* rapid reinfection following treatment as well as the cognitive benefits of natural declines in STH infections among school-aged children. By modeling the relationship between helminth infections and cognitive testscores from the interval of least infection following *S. japonicum* treatment, we highlight the cognitive test performance advantage of sustained low level single and polyparasitic helminth infections that is derivable in the presence of systematic frequent deworming programs. This relationship may be blunted or lost in an environment characterized by infrequent deworming and high helminth reinfection pressure. Findings from this design and analytic strategy may be more generalizable to the actual implementation of deworming programs than randomized trials.

We conclude that declines in the burden of some helminth species and polyparasitic STH infections have beneficial long-term impacts on children's cognitive performance. Our results highlight the benefit of combined control for *S. japonicum* and STH infections; it further stresses the importance of sustained deworming for improving the learning, memory, and educational attainment of children in helminth-endemic settings. The benefit of combined treatment for these infections notwithstanding, deworming is only a necessary first step in the implementation of a comprehensive integrated helminth control program, which must be tailored to a given endemic setting and include provision of clean water and improved sanitation to mitigate the fundamental causes of these infections and their associated adverse health effects among the most vulnerable populations [43], [44].

## Supporting Information

Appendix S1 **Choice of cognitive tests, rationale for test choice, and the psychometric properties of respective tests.**
(DOC)

Appendix S2 **The Philippine nonverbal intelligence test – a further description.**
(PDF)

Appendix S3 **Details of multivariable regression models used in statistical analyses.**
(DOC)
