# Supplementary material for: Treatment for Schistosoma japonicum, Reduction of Intestinal Parasite Load, and Cognitive Test Score Improvements in School-Aged Children
Source: PLoS Negl Trop Dis. 2012 May 1;6(5):e1634. doi: 10.1371/journal.pntd.0001634 (PMC3341324; doi:10.1371/journal.pntd.0001634)
Supplement: Appendix S1 — Choice of cognitive tests, rationale for test choice, and the psychometric properties of respective tests. (DOC) [file pntd.0001634.s001.doc]

**APPENDIX S1: COGNITIVE TESTS**

**Rationale for Choosing Particular Tests:** The choice of tests used in this study is based both on practical and theoretical considerations. The practical considerations address some of the difficulties inherent in administering tests to children in the developing world that were constructed with American children. Baddeley holds that neuropsychological tests developed in the US and Europe may be effectively adapted for use in the developing world, [1] particularly if certain considerations are addressed: (i) tests are culturally and linguistically adaptable for use in developing countries; (ii) tests have been shown to be reliable in the developing world; (iii) tests are easy to administer even under often non-ideal field conditions; and (iv) tests are sensitive to differences between individuals within a given culture, but do not need to provide valid comparisons across cultures. Our choice of tests (described in detail below) meet these requirements.

Tests were chosen that assess domains of cognitive function which have been previously found to be sensitive to changes in the primary exposures addressed in this proposal. Thus, *Verbal Fluency* was chosen since deficits in the cognitive domains assessed by this test (long term memory) was found to be causally related to *S. japonicum* infection in a Chinese study conducted by Nokes et al. [2] Further, pro-inflammatory cytokines have been related to specific cognitive deficits in long- and short-term memory [3]. The *Wide Range Assessment of Memory and Learning* (*WRAML*) was chosen because it addresses other domains of memory and provides a summary index of memory which addresses a range of memory functions. The WRAML was also chosen because it contains three sub-tests that assess learning. Impact on learning ability represents a potentially long-term influence of parasitic infections in childhood. Assessment of learning abilities emphasizes outcomes relevant to children’s daily functioning as well as the long term rewards of education[4].

**Reliability Assessment** We translated these tests, adapted them for use in The Philippines, and established their inter-rater reliability (see preliminary studies). We also assessed test-retest reliability and the validity of these tests in this population of rural Filipino children. Validity was assessed by comparing test results to overall school achievement for the semester in which the test is administered, as a form of construct validation. In addition, for the WRAML, conducted a principal components analysis using results from its subscales and a two factor varimax rotation to ensure that the three subscales within each of the two domains (verbal memory and learning) loaded onto its respective domain.

**Test Administration** Cognitive function tests were administered by individuals from The Philippines who speak the local languages spoken among the children to be tested (Tagalog and Waray). They were been trained by Dr. Friedman to implement respective tests. These individuals: i) were well educated (each with a minimum of bachelor’s degree); ii) had previous experience working with children; iii) and demonstrated to the investigators an ability to establish good rapport with children.

Test administration for children recruited into the study occurred in a quiet room without distractions in a local school on a weekend day. An attempt was made to assess each child at the same time of day on each occasion. Each child was provided with a small snack before testing as hunger has been shown to adversely affect performance on these tests[5]. The rater spent about 5 minutes talking to each child before testing began and provided praise for the child’s effort throughout testing to encourage their active participation[1].

**Specific Tests** Based on the considerations outlined above, the following tests were used:

**1) *Verbal Memory and Learning Indices of the Wide Range Assessment of Memory and Learning (WRAML)***[6]: This set of nine subtests evaluates a child’s ability to actively learn and memorize a variety of information. Adaptation of the three visual memory subtests that comprise the Visual Memory Index for use among Filipino children proved difficult. We, therefore, used only the three verbal memory subtests that comprise the Verbal Memory Index and the three learning subscales that comprise the Learning Index. Scaled and standard scores allow performance comparisons based on age. We will thus be able to derive an age standardized score for each volunteer, which is important as volunteers will age over the 18-month follow-up period. Specific subtests of the WRAML have been validated against school performance and have been shown to be significantly related to academic achievement in the subject areas they most closely mimic. Finally, the WRAML was adapted and successfully used in a developing world population in Malaysia[7]. We used the Verbal Memory and Learning Indices as our primary outcome measures for cognitive function.

**2) *Verbal Fluency:*** This test has been used in a multitude of studies in the developing world, where it has been shown to be reliable across a variety of field settings [1,2]. It is easy to explain and is described as an “acceptable task across a wide range of cultures”[1]. The child names as many items in a given category as he/she can in 60 seconds after a practice category. Verbal fluency captures working memory function [1,2].

***3) Philippine nonverbal intelligance test (PNIT):*** The PNIT is an intelligence test that measures concept recognition and abstract thinking[8]. We obtained this test from Dr. Guthrie who developed the test in The Philippiines in the 1970's. We were unable to find it commercially or through other means. We include the original publication from Dr. Guthrie which has more details about this test. This test was used because it was developed in The Philippines and its properties have been described in this population.

**References**

1. Baddeley A, Gardner JM, Grantham-McGregor S (1995) Cross-cultural Cognition: Developing Tests for Developing Countries. Applied Cognitive Psychology 9: S173-S195.

2. Nokes C, McGarvey ST, Shiue L, Wu G, Wu H, et al. (1999) Evidence for an improvement in cognitive function following treatment of Schistosoma japonicum infection in Chinese primary schoolchildren. Am J Trop Med Hyg 60: 556-565.

3. Reichenberg A, Yirmiya R, Schuld A, Kraus T, Haack M, et al. (2001) Cytokine-associated emotional and cognitive disturbances in humans. Arch Gen Psychiatry 58: 445-452.

4. Drake LJ, Jukes MCH, Sternberg RJ, Dundy DAP (2000) Geohelminth infections (Ascariasis, Trichuriasis, and Hookworm): cognitive and developmental impacts. Seminars in Pediatric Infectious Diseases 11: 245-251.

5. Simeon DT, Grantham-McGregor S (1989) Effects of missing breakfast on the cognitive functions of school children of differing nutritional status. Am J Clin Nutr 49: 646-653.

6. Sheslow D, Adams W (1990) Wide Range Assessment of Memory and Learning: Administration Manual; Inc. WR, editor.

7. Ong LC, Chandran V, Zasmani S, Lye MS (1998) Outcome of closed head injury in Malaysian children: neurocognitive and behavioural sequelae. J Paediatr Child Health 34: 363-368.

8. Guthrie GM, Tayag AH, Jimenez-Jacobs P (1977) The Philippine nonverbal intelligence test. Journal of Social Psychology 102: 3 - 11.
