# Supplementary material for: Treatment for Schistosoma japonicum, Reduction of Intestinal Parasite Load, and Cognitive Test Score Improvements in School-Aged Children
Source: PLoS Negl Trop Dis. 2012 May 1;6(5):e1634. doi: 10.1371/journal.pntd.0001634 (PMC3341324; doi:10.1371/journal.pntd.0001634)
Supplement: Appendix S2 — The Philippine nonverbal intelligence test – a further description. (PDF) [file pntd.0001634.s002.pdf]

The Journal of Social Psychology, 1977, **102**, 3-11.

THE PHILIPPINE NONVERBAL INTELLIGENCE TEST*[1]

Department of Psychology, The Pennsylvania State University; Philippine Normal College, Manila, Philippines; and Indiana, Pennsylvania

GEORGE M. GUTHRIE, AMANDA H. TAYAG, AND PEPITA JIMENEZ JACOBS

SUMMARY

Problems arising from the cultural bias of intelligence tests can be reduced by developing new tests within the society in which they are to be used. With the use of an item format in which *S* points to one of five illustrations which does not belong with the other four, a nonverbal test of 100 items was developed by Filipinos for Filipino children. Standardized on 1200 school children in the Philippines, the test showed high reliability and satisfactory validity. Results indicate that different norms are needed for urban and rural children. The format readily permits new items and a new analysis when new cultural groups are tested.

A. INTRODUCTION

In this paper we will report the development and standardization of an intelligence test in a non-Western society, the Philippines, and the assessment of its reliability and validity. Filipinos recognize, as do members of other societies, that some individuals are *marunong* or smart, that they learn more rapidly than their peers, and that such differences are most apparent in schools. These were the circumstances that led to Binet's pioneering work in France and to many of the applications of tests at the present time in North America and Western Europe. But, while societies the world over recognize the existence of differences in intellectual abilities, these differences are expressed in behavioral capacities, particularly verbal, which are quite dissimilar from one society to another. Translation of questions from one language to another does not make it possible to

* Received in the Editorial Office, Provincetown, Massachusetts, on May 12, 1976, and given special consideration in accordance with our policy for cross-cultural research.
Copyright, 1977, by The Journal Press.
[1] The authors wish to thank Felisa Librea Faminiano for her contributions as the artist who drew the items and who conceived many of them as well. The final draft of this paper was completed while the first author was a Senior Fellow at the Culture Learning Institute, East-West Center, Honolulu, Hawaii.

Copyright © 2001. All Rights Reserved.

transfer tests because questions are deeply rooted in the experiences of the members of the society in which the test was formulated. Even many nonverbal test materials are experienced differently due to such factors as instructions or speed of response requirements.

Marked differences among societies in the expression and content of intellectual processes have made it impossible to compare intellectual levels from one society to another. The social implications of declaring members of one society more intelligent on the average than those of another society have also been recognized and have led in some quarters to a rejection of any measurements of ability even within a given group. Differences in intellectual performance are, however, found in all societies; those who perform well contribute greatly to the welfare of their society, while the poorest performers must be cared for because they cannot care for themselves.

There are hazards in the use of measures of intelligence for both groups and individuals, since some may be labelled and then treated as inferior, creating a self-fulfilling prophecy. These negative consequences are not necessary if tests are used and interpreted by professional people who understand the cultural specificity of test items for groups and the unreliability of individual tests, as well as the possibility of various remedial programs for individuals who are developing slowly. Where educational resources are limited, tests may help to find those most likely to benefit, a selection procedure which may be fairer than selection based on family influence or teachers' biases. In addition, other cultures provide natural laboratories for the study of the effects of child rearing practices, nutrition, linguistic factors, and socioeconomic and school conditions on intellectual development. For instance, the differences between urban and rural environments as they may bear on cognitive development appear to be much greater in developing than they are in industrialized countries.

In their attempts to assess intellectual development in societies other than their own, psychologists have relied heavily on nonverbal tests, such as the Porteus Mazes, Raven Progressive Matrices, Goodenough-Harris Drawing Test, and the Cattell Culture-Fair Tests. Relatively few attempts have been made to develop tests within the society in which the testing was to be done. A notable exception is the adaptation and standardization of tests for use in New Guinea by Ord and others (12). In these attempts to measure intelligence it has been learned that a mere translation of instructions and questions is not enough. The child's performance can be distorted also by the unfamiliarity of the materials, the imposition of time limits, the

Copyright © 2001. All Rights Reserved.

insistence that the child work alone without help from peers or testers, and by the fact that a correct answer is demanded rather than some other interaction, such as cooperative play or the imitation of an adult. Problems of adapting tests and of testing in other cultural settings have been discussed by Vernon (18), with applications in Jamaica and Uganda and with Canadian Indians and Eskimos. Somé strategies which may be used in test construction have been discussed by Ortar (13) and Cortada de Kohan (3) and others in the useful conference report by Cronbach and Drenthe (5) which dealt with mental tests and cultural adaptation. Irvine (10) has provided a broader perspective on the assessment of intellectual development in other societies.

Much has been learned that is useful to test constructors in studies of concept formation and in the application of Piaget's procedures with non-Western *S*s. These efforts were prompted in part to challenge the assertions of Levy-Bruhl (11) and many others that so-called primitives were incapable of abstract thought. Early work with psychological tests tended to confirm poor abstracting abilities until Price-Williams (15) demonstrated that Nigerian literate and illiterate children could carry out abstract classifications if the *E* used familiar indigenous materials, such as local animals or plant forms. Gay and Cole (6) have extended the use of indigenous materials in a study of the quantitative behavior of the Kpelle, a tribal group in Liberia and later (2), in an examination of classification, learning, and memory. There is a dilemma, however, if one wishes to compare the performance of members of two or more societies. If we keep the testing materials constant from one society to another, differences in results may be attributable to differences in familiarity with the materials, but if we change materials from one society to another to deal with the familiarity problem we cannot be certain that tests are equivalent.

The foregoing considerations apply to measurement attempts as much as to experimental tasks in research on cognitive processes. A solution would appear to be to develop an intelligence test within a culture and then to carry out a series of studies which show that the scores are related to various indicators of intelligent behavior. Such a procedure could yield converging evidence of the validity of the test in a situation where other criteria for validation are not available.

B. Method

In light of the foregoing results, the developer of a test of intelligence within a society must pay attention to the task, the instructions, the

Copyright © 2001. All Rights Reserved.

materials, and the social relationship between tester and S. Specifically, in the Philippines where many different languages are spoken one would prefer a nonverbal test with simple instructions which do not change frequently as the test progresses, and a test composed of objects familiar to Philippine children. Standardization of items and development of norms would be entirely within the Philippines, and the criteria of validity would also be obtained within the Philippines.

In order to meet the conditions outlined above we adopted a format of a series of classification tasks in which the S has to develop a concept which will encompass four of the alternatives and exclude a fifth. In this style of test item we followed the Columbia Mental Maturity Scale (1). This is a familiar type of item in many tests and a commonly used method of studying concept formation. Although the easiest items involve merely perceptual similarity, by the sixth item an unspoken abstraction is necessary, such as "four of the five are birds," and by the twelfth, "four are fruits, the other a vegetable." Furthermore, the basis of classification shifts with every item. Items with two or more equally satisfactory solutions were eliminated during extensive pretesting. The item analysis enabled us to make sure that the mean score of those who chose the alternative keyed correct was higher than the means of those choosing any of the four wrong alternatives. Using this format we were able to call upon our Ss to develop increasingly abstract categorizations, and to shift concepts from one item to the next. But neither the examiner nor the S was required to explain the abstractions. We were able to assess aspects of implicit verbal reasoning behavior without the explicit use of language.

The Philippine Nonverbal Intelligence Test (PNIT), developed in the Philippines, is a 100-item test for children five to 14 years of age (8). On a separate card, 9 cm × 33 cm, each item presents drawings of five objects, designs, or numbers and the S is asked in his home language or in pantomime "Which one is different, which one does not go with the others?" The S is required only to point to the one which is different; he is not asked to explain his choice. The first items are very simple concrete perceptual tasks which include four arrows pointing up, one down; four circles and one solid black; and four squares of varying sizes and a circle. These early, very simple items help the S to learn and master the task. The instructions need to be repeated for only the first two or three items. The items are arranged in order of difficulty with the basis of classification becoming increasingly abstract. The content shifts from one item to the next so that a new basis of classification has to be developed by the S for

Copyright © 2001. All Rights Reserved.

each item. There is no time limit on each item; the whole test takes 20 to 30 minutes as a rule. The score is the total number correct with no correction for guessing.

Several decisions reflected in the above paragraph are expressions of Philippine cultural patterns. A time limit, for instance, was not only undesirable but unnecessary because Philippine children responded very quickly; often too quickly to permit careful examination of the item. This reflects the relative subjective costs to a Philippine child of implying that he does not know by delay or by failure to respond *versus* making a mistake. The children learn in school, and in their homes as well, that one appears stupid if he delays too long but not stupid if he is wrong but prompt. The result is that only a small number of times in thousands of testing sessions has a child stopped because items are too difficult. On the contrary, while children move from a higher to a lower level of accuracy as they progress, the latency of their response does not change. Neither child nor tester gives any indication that he is just getting chance of one answer correct in five. For these reasons it was considered unwise to terminate the test after a specified number of errors. A similar phenomenon has been reported (7) where Philippine college students would guess without delay, leaving no items blank, on a difficult multiple choice test of reasoning. Almost all *S*s finished the test well before the time limit but total scores were very close to chance. The decision not to ask for the basis of classification or for any verbal response was based on the fact that some Philippine children would be very shy about talking to a tester and also on the fact that, with many languages, there would be frequent instances of failure to understand the *S*'s response.

The test was developed by the three authors and the artist who was also a teacher. Each item was tried out informally on friends and on children of the Laboratory School of the Philippine Normal College. Promising items were collected, arranged in judged order of difficulty, and administered to a sample of several hundred students. The 120 items in the original pool were reduced to 100 by dropping those which failed to correlate highly with the total score, which were too difficult or too easy or which had two or more alternatives that appeared equally as accurate as the alternative keyed as correct by the authors.

Final standardization was carried out at a large public elementary school in Manila where data were collected on 100 boys and girls at each grade level. Analysis of these data was made simple by using a computer program developed to evaluate five alternative multiple choice tests for college

Copyright © 2001. All Rights Reserved.

students. The computer program produced a comprehensive analysis of the test, providing overall reliability estimates and an item-by-item analysis which indicated the discriminating power of each item, the attractiveness of each wrong alternative, and the mean score of those who chose each alternative, right or wrong. By recording the *S*'s responses on the machine-scored answer sheets used with college students we were able to proceed from data collection to analysis with no intermediate tabulation or transfer of data. These analyses were performed in the United States where computer facilities were available at that time.

C. Results

The test which we constructed following the procedures outlined above proved to have many properties which are considered desirable, all of which were determined against criteria obtained in the Philippines. Kuder Richardson Formula 20 reliability estimates ranged from .71 to .95 at six grade levels in a rural school and from .77 to .90 with eight samples of children in public and private schools of Manila.

The mean raw scores showed a steady progression of about five items from one grade level to the next but there were marked differences between urban and rural populations. Urban public school children in the early grades were about one grade level higher than rural children, a difference which reached two grade levels by the sixth year of elementary school. But we found consistently that there were no differences in means between boys and girls. Finally, the PNIT was designed and standardized as an individually administered test. We have attempted to administer it in groups with each student recording his own choice of alternatives but this method led to means lower than expected and to a substantial drop in KR20 estimates of reliability.

The validity of the test was assessed against the grades which students had received in various courses. The results, shown in Table 1, indicate that validity coefficients obtained with this test are about the same as those obtained with American tests given to American children. While the coefficients are low, one must bear in mind that the criteria are of low reliability and that factors in addition to intellectual ability influence classroom performance.

Norms have been developed for three populations of school children which include 815 enrolled in the Elementary Laboratory School of the Philippine Normal College in Manila; a careful sample of 140 children at each of six grade levels, for a total of 840, in the public schools of Baguio, a

Copyright © 2001. All Rights Reserved.

TABLE 1
CORRELATIONS BETWEEN THE PHILIPPINE NONVERBAL INTELLIGENCE TEST AND GRADES

Sample Manila schools	*N*	School *S*s: Reading	Language	Mathematics	Social Studies
Grade II					
Public school					
Boys	102	.21*	.30**	.36**	.34**
Girls	98	.04	.04	.11	.00
Private school					
Boys	101	.34**	.44**	.37**	.00
Girls	99	.33**	.28**	.29**	.30**
Grade V					
Public school					
Boys	98		.41**	.28**	.17
Girls	96		.28**	.28**	.21
Private school					
Boys	98	.26**	.28**	.41**	.24*
Girls	96	.33**	.32**	.45**	.34**
Rural school					
Grade I	74		.14	.17	.21
Grade II	60		.16	.24*	.10
Grade III	54		.21	.18	.24*
Grade IV	85		.38**	.28*	.26*
Grade V	84		.05	.11	.28*
Grade VI	55		.46**	.61**	.57**

* $p < .05$.
** $p < .01$.

small city in the mountains north of Manila (17); and 800 town and 400 rural children from the rice plain north of Manila. In each case we have interpreted the scores in terms of percentiles for grade placement and for age groupings. In order to emphasize the within-sample comparative purpose which we have in mind for the test, we have not developed one set of norms nor do we propose a nationwide or broad sampling. Within limits, each user should develop his own norms or use those from the group which most closely resembles his population. The test was designed to provide a measurement of how a given student compared with others in his community and not to compare one group of children with another group from a different social setting.

The PNIT has been used to assess the relationship between indices of malnutrition and intellectual development (9) and to determine whether an injection to reduce iodine deficiency resulted in improved intellectual growth, as well as in reduced thyroid size (14). It has also been used with children in remote villages where the range of scores and apparent comprehension of the task suggest that satisfactory measurement is possible. It has also been used extensively in East Malaysia or Borneo by Seymour (16)

Copyright © 2001. All Rights Reserved.

who reported similar high estimates of reliability and higher validity coefficients against teachers' ratings and grades than we report for the Philippines. Seymour did alter a half dozen items to make the material presented more familiar to Malaysian children.

While further research is necessary to determine more precisely which components of intelligence are being assessed by the PNIT, the format and content of the items, as well as the validity coefficients, indicate that, following Cronbach's proposed spectrum of abilities (4, p. 282), it should be considered a measure of analytic or "fluid" ability rather than "crystalized" achievement.

It is the intent of the authors that new norms be collected when the PNIT is given to groups markedly removed in culture and environment from the Filipino children on whom it was standardized. In this step a researcher might choose to drop or modify items which pretesting shows to be ambiguous or meaningless to his *S*s. When several hundred *S*s are tested a new item analysis should be considered, a step that is now well within the realm of possibility, given the widespread distribution of computing facilities.

References

1. Burgmeister, B. B., Blum, L. H., & Lorge, I. Columbia Mental Maturity Scale. Yonkers-on-Hudson, N. Y.: World Book, 1954.
2. Cole, M., Gay, J., Glick, J. A., & Sharp, D. W. The Cultural Context of Learning and Thinking. New York: Basic Books, 1971.
3. Cortada de Kohan, N. Test construction and standardization in different cultural settings. In L. J. Cronbach & P. J. D. Drenthe (Eds.), *Mental Tests and Cultural Adaptation*. The Hague: Mouton, 1972. Pp. 121-127.
4. Cronbach, L. J. Essentials of Psychological Testing (3rd ed.). New York: Harper & Row, 1970.
5. Cronbach, L. J., & Drenthe, P. J. D. Mental Tests and Cultural Adaptation. The Hague: Mouton, 1972.
6. Gay, J., & Cole, M. The New Mathematics and an Old Culture. New York: Holt, Rinehart & Winston, 1967.
7. Guthrie, G. M. Structure of abilities in a non-Western culture. *J. Educ. Psychol.*, 1963, **54**, 94-103.
8. Guthrie, G. M., Tayag, A. H., & Jacobs, P. J. The Philippine Nonverbal Intelligence Test. Manila: Philippine Normal College, Child Study Center, 1968.
9. Guthrie, H. A., Guthrie, G. M., & Tayag, A. H. Nutritional status and intellectual performance in a rural Philippine community. *Phil. J. Psychol.*, 1968, **1**, 28-34.
10. Irvine, S. H., & Carroll, W. K. Testing and assessment across cultures: Issues in methodology and theory. In H. C. Triandis (Ed.), *Handbook of Cross-Cultural Psychology*. In press.
11. Levy-Bruhl, L. How Natives Think. London: Allen & Unwin, 1926.
12. Ord, I. G. Mental Tests for Pre-Literates. London: Ginn, 1970.
13. Ortar, G. Some principles for adaptation of psychological tests. In L. J. Cronbach & P.

Copyright © 2001. All Rights Reserved.

J. D. Drenthe, *Mental Tests and Cultural Adaptation*. The Hague: Mouton, 1972. Pp. 111-120.

14. PADLAN, A. A., & GUTHRIE, G. M. Iodine supplementation and intelligence in school children. In preparation.
15. PRICE-WILLIAMS, D. R. Abstract and concrete modes of classification in Nigeria. *Brit. J. Educ. Psychol.,* 1962, **32**, 50-61.
16. SEYMOUR, J. M. The rural school as an acculturating institution: The Iban of Malaysia. *Hum. Org.,* 1974, **33**, 277-290.
17. TORRENTO, C. J., & NGALOB, J. Establishing norms for the Philippine Non-Verbal Intelligence Test in Baguio City's elementary schools. Unpublished Master's thesis, Saint Louis University, Baguio, Philippines, 1971.
18. VERNON, P. E. Intelligence and Cultural Environment. London: Methuen and Company, 1969.

Department of Psychology
The Pennsylvania State University
417 Moore Building
University Park, Pennsylvania 16802

Copyright © 2001. All Rights Reserved.
