# Supplementary material for: Treatment for Schistosoma japonicum, Reduction of Intestinal Parasite Load, and Cognitive Test Score Improvements in School-Aged Children
Source: PLoS Negl Trop Dis. 2012 May 1;6(5):e1634. doi: 10.1371/journal.pntd.0001634 (PMC3341324; doi:10.1371/journal.pntd.0001634)
Supplement: Appendix S3 — Details of multivariable regression models used in statistical analyses. (DOC) [file pntd.0001634.s003.doc]

APPENDIX S3

Multivariable Regression Models:

1. Estimating effect of 1) S. japonicum decline or 2) polyparasitic STH decline on testscores – Both exposure and outcome are time varying in this model.

E(Testscore_it_)=β_0_+β_1_time_it_ + β_2_Δ*S. japonicum*_it_ + β_3_Δ*S. japonicum*_it_**TIME*_i_ +β_4_ΔSTH_it_ + β_5_Baseline_*S. japonicum* +β_6_ Baseline_ sth+β_7_Age@enrollment + β_8_sex+β_9_ses + β_10_Baseline Underweight +β_11_Baseline_Anemia

For *S. japonicum* only, estimating differences in testscores based on timing of reinfection – note that the exposure is not time-varying within individuals in this model. Only the outcome (Testscore) is.

E(Testscore_it_)=β_0_+β_1_time_it_+β_2*_*S. japonicum_reinfection_interval*_i_ +β_3_Baseline_*S.japonicum* + β_4_Baseline sth +β_5_Age@enrollment+ β_6_sex +β_7_ses + β_8_Baseline_Underweight+β_9_Baseline_Anemia

Where: *S. japonicum-reinfection-interval*_i_ is a 4 level categorical variable defined as follows:

0 if not re-infected by month 18;

1 if re-infected between months 12 and 18;

2 if reinfected between months 6 and 12 and

3 if never cured or *S. japonicum* positive in t_1_, t_2_ and t_3_ (reference group).
